# Phenolics as ecologically relevant cues for slime flux breeding *Drosophila virilis*

**DOI:** 10.1016/j.isci.2024.111180

**Published:** 2024-10-18

**Authors:** Venkatesh Pal Mahadevan, Regina Stieber-Rödiger, Markus Knaden, Bill S. Hansson

**Affiliations:** 1 Department of Evolutionary Neuroethology, Max Planck Institute for Chemical Ecology, Jena, Germany; 2 Max Planck Center next Generation Insect Chemical Ecology, Max Planck Institute for Chemical Ecology, Jena, Germany

**Keywords:** Entomology, Biochemistry, Sensory neuroscience, Evolutionary biology

## Abstract

*Drosophila* species belonging to the *virilis* group offer a unique opportunity for studying olfactory adaptations necessary for survival within forest ecosystems as many of these species breed within decaying plant vascular tissues. However, the knowledge regarding olfactory preferences within their ecological niche is extremely limited. Here, we focus on *Drosophila virilis* and identify over 120 distinct odors from a natural slime flux source. We identify lignin as an attractant and a positive oviposition cue for *D. virilis.* Furthermore, lignin-derived guaiacol is highlighted as a robust attractant for *D. virilis*. We demonstrate that guaiacol is detected by the *Dvir*Or49b receptor, which exhibits a narrow sensitivity to methylphenols, including *o*-cresol. *D. virilis* and *D. ezoana*, both belonging to the *virilis* group, exhibit strong attraction to *o*-cresol. In summary, our research offers a comprehensive analysis of the diverse array of odorants encountered by *D. virilis* within its natural habitat and their behavioral significance.

## Highlights

• The study reports the chemical analysis of a slime flux sample
• Lignin and its volatile derivative guaiacol act as strong attractants for *D. virilis*
• Guaiacol along with *o*-cresol is detected by *Dvir*Or49b
• *o*-cresol, found in the beaver castoreum, is a strong attractant for *D. virilis*

## Graphical abstract

**Figure:**
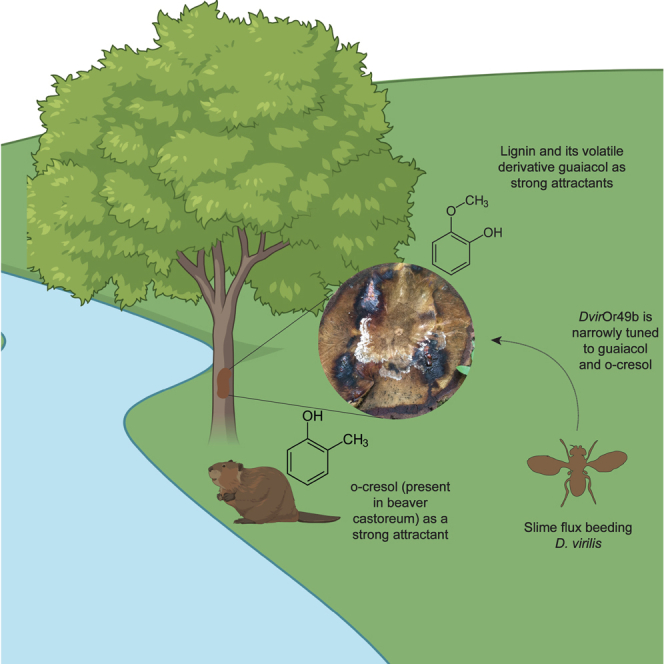

## Introduction

Colonization and adaptation to new ecological niches represent a defining characteristic of the speciation process.^1^ This phenomenon is highly relevant for animals heavily reliant on their sense of smell, as they must undergo adjustments to recognize and respond to the volatile cues specific to their newly inhabited environment. Such adaptations frequently drive evolutionary modifications in peripheral olfactory coding systems, offering an opportunity for researchers to unveil the underlying mechanisms of speciation.

The genus *Drosophila* stands out as an exceptional model for studying these processes, given its well-documented capacity to inhabit a wide range of ecological niches.^2^^,^^3^^,^^4^^,^^5^^,^^6^^,^^7^^,^^8^ Among the extensively studied *Drosophila* species, categorization is often based on their specific niches and dietary preferences, encompassing categories such as saprophagous, mycophagous, flower-breeding, herbivorous, and cactophagous drosophilids, to name just a few.

Within this diverse spectrum, saprophagy entails the breeding and feeding on decomposing organic matter. Of special interest is the observation that numerous *Drosophila* species have been documented to reproduce and feed on decaying tree barks, primarily sustained by yeasts associated with the decomposition of tree vascular tissues.^9^^,^^10^^,^^11^^,^^12^^,^^13^ Trees experiencing injury or those releasing nutrient-rich exudates during early spring emerge as attractive hosts for a plethora of microbial species. Subsequent inoculation and microbial growth contribute to the formation of what is known as bacterial wet wood, colloquially referred to as slime flux.^10^^,^^14^

Species belonging to the *virilis* group, a subset within the *Drosophila* subgenus, have undergone specialized adaptations to inhabit such an ecological niche characterized by the presence of slime flux from various temperate tree species such as birch, aspen, willow, and others.^11^^,^^12^^,^^15^ Despite its intriguing nature, however, there exists a significant gap in our understanding of the natural dynamics of this interaction, particularly concerning the olfactory aspects. This knowledge void can be attributed to two principal factors. Firstly, some of the early observations of *Drosophila*-slime flux interactions in the 1950s were conducted not by ecologists but rather by mycologists who were primarily focused on studying the microbial communities within these fluxes.^9^ Secondly, the occurrence of slime fluxes, which actively attract insects, is often challenging to predict, and these occurrences can be concealed within the intricate structure of tree bark.^9^^,^^11^^,^^12^ As a result, a comprehensive understanding of the intricate bouquet of natural odors emanating from these slime fluxes, and correspondingly, the specific olfactory cues employed by *Drosophila* species that breed within these fluxes to locate suitable breeding sites, remains largely uncharted territory.

Our primary objective was to elucidate the olfactory background to the interaction between flies belonging to the *virilis* group and slime flux. To achieve this, we selected *Drosophila virilis* as our focal species. *D. virilis* has a cosmopolitan distribution and a broad dietary spectrum among decaying tree matter and slime fluxes.^16^

We collected and identified over 120 chemically diverse odor compounds from a slime flux. Among these compounds, guaiacol, a breakdown product of lignin present in natural slime flux, emerged as a potent attractant for *D. virilis*. In subsequent investigations, we established that the *Dvir*Or49b receptor is responsible for detecting guaiacol, displaying a narrow sensitivity to phenolic compounds, including *o*-cresol. *o*-cresol has been reported in nature as a constituent of beaver castoreum.^17^ This discovery raises intriguing possibilities, considering the hypothesis of an indirect commensal relationship between beavers and species within the *virilis* group,^18^ where the beaver’s environmental engineering would provide suitable food sources and breeding sites for the flies. Lastly, after screening a number of *Drosophila* species, our research unveiled *o*-cresol as a robust attractant specific to *D. virilis* and *D. ezoana*.

In summary, our study sheds light on odor-driven behaviors within the ecological context of *D. virilis*, thereby providing crucial insights into the intricacies of this species’ interactions with its environment.

## Results and discussion

### Slime flux collection

**Figure 1:**
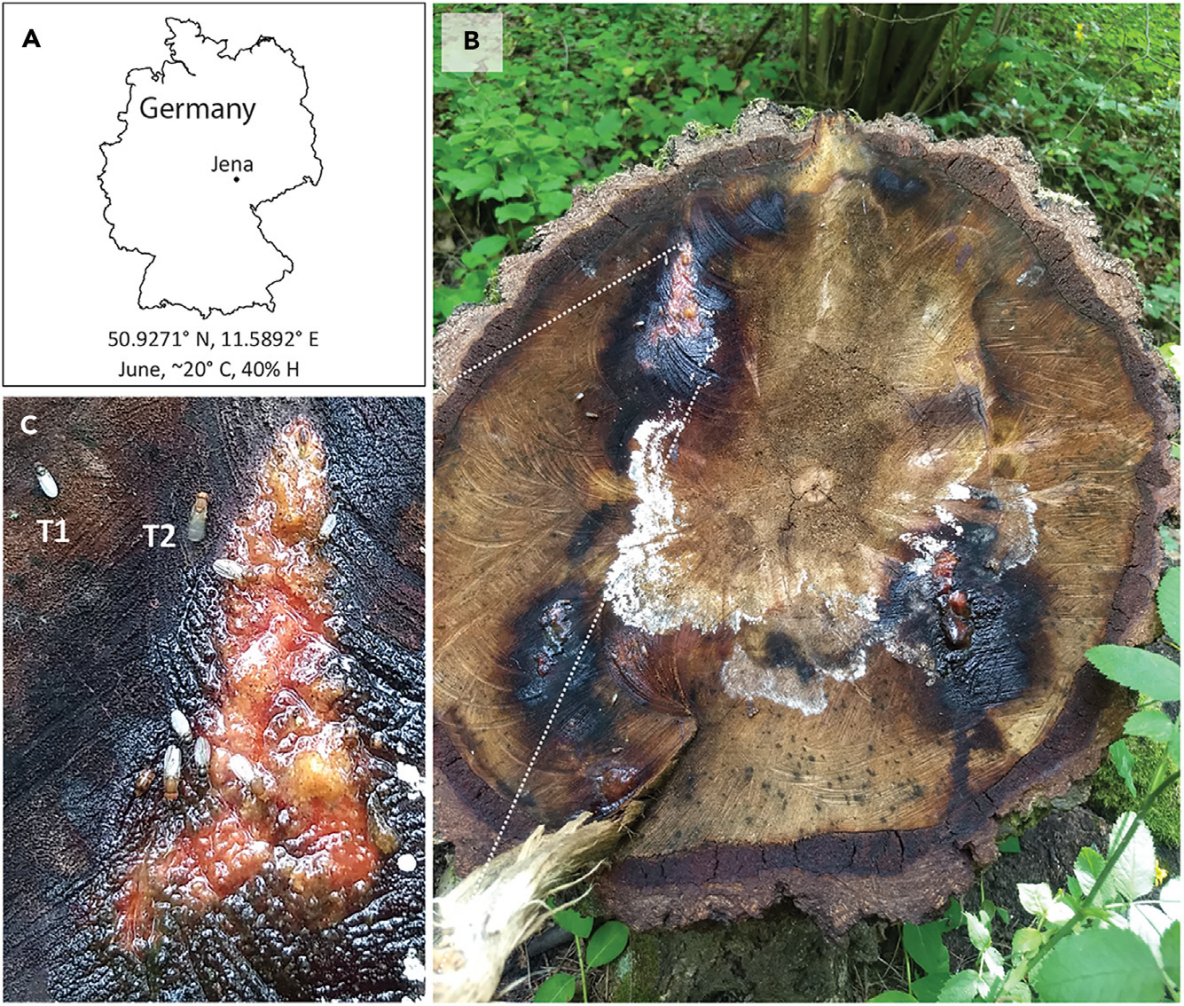
Slime flux collection site (A) Location of the slime flux collection site in Germany. (B) A cut birch tree producing slime flux where three distinct slime flux spots can be observed. (C) Two morphologically distinct types of drosophilids (T1 & T2) were observed to feed from the slime flux. Larvae were observed in the slime flux, while other insect species were not taken into account (photo by: V.P.M.).

The *virilis* group comprises twelve species that are known for their association with slime flux.^9^^,^^15^ These species are predominantly distributed across the temperate region of the world with their origin predicted to be from Asia.^15^ Typically, being monophagous in nature, these species (except for *D. virilis*) are known to favor rotting vascular tissues of birch, willows, and cottonwood as their natural hosts.^18^^,^^19^^,^^20^ Moreover, several other *Drosophila* species such as *D. robusta* and *D. pseudoobscura* (not belonging to the *virilis* group) are also known to be associated to breed from slime fluxes.^9^^,^^10^^,^^13^ However, the volatiles associated with such fluxes that would have an ecological relevance in these species have still not been described. In this study, we first set out in search of an active slime flux intending to explore volatiles associated with it. The search was carried out in the forests around Jena, Germany, starting from spring to early summer (mid-March to mid-June 2020–21) (Figure 1A). In these forests, tree species such as birch, aspen, willow, etc., are prevalent. Although most of the forest was in sound condition, we serendipitously came across a cut, birch tree (Figure 1B), which was actively producing slime flux during early June (∼20°C, 40% humidity). Moreover, the site was actively visited by multiple insect genera comprising at least two morphologically distinct *Drosophila* species (Figure 1C, T1&T2) with larvae visible in the slime. Flies were collected from the site using manual aspiration. However, molecular identification revealed the presence of *D. immigrans* and *D. pseudoobscura* at the sap sites. Previous reports have suggested an association between slime flux and *D. pseudoobscura*, while the presence of *D. immigrans*, a known human commensal, could just be a virtue of human housings based at the foothill.^13^^,^^21^ Slime samples were collected and their hexane/methanol extracts were stored at −20°C until subsequent chemical analysis.

### The chemistry of slime flux reveals phenolics as ecologically relevant cues for *D. virilis*

**Figure 2:**
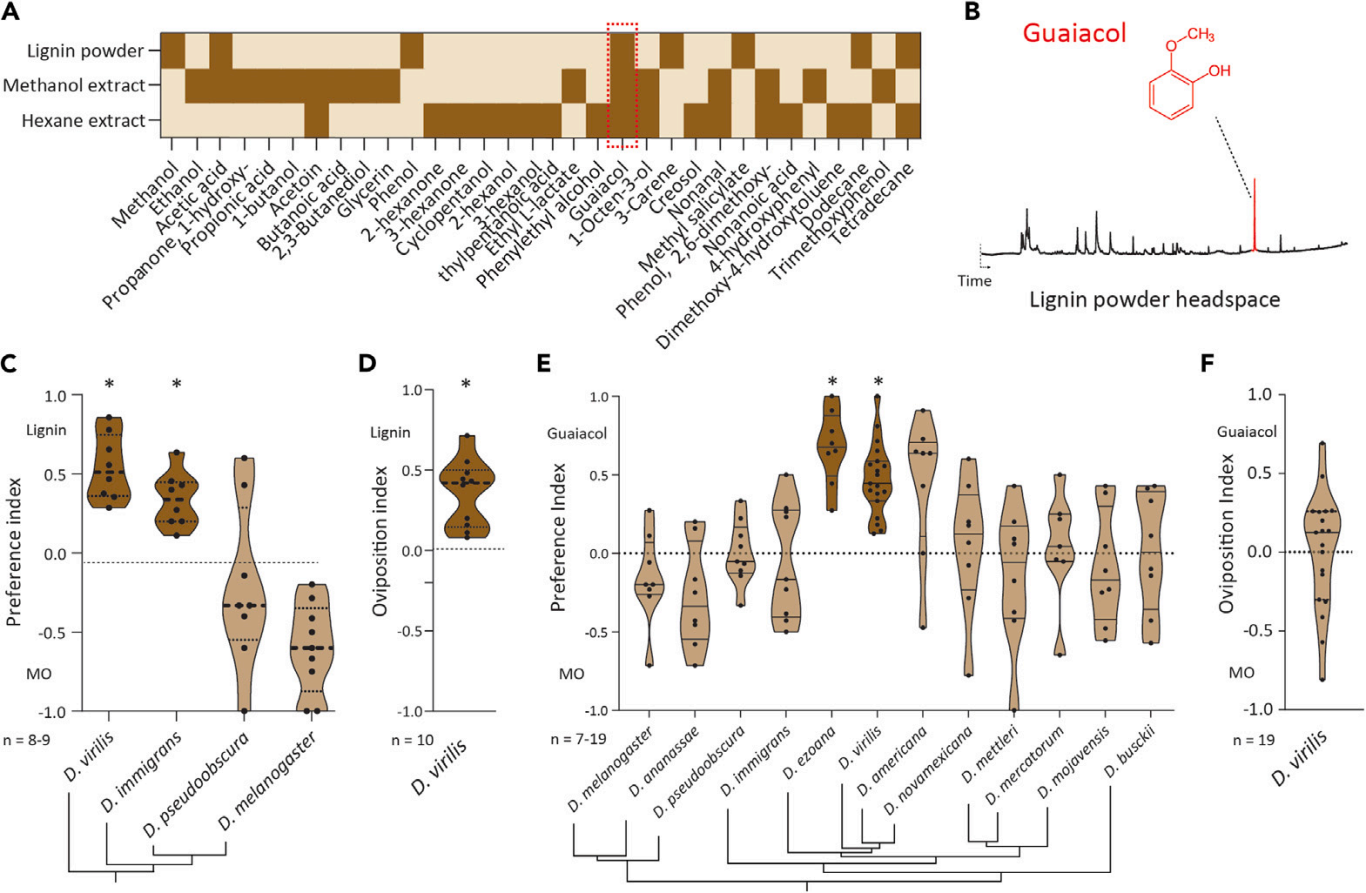
Volatile phenolic derivatives as attractants for *D. virilis* (A) Heatmap representing data obtained from the analysis of chromatogram traces obtained from multiple stimuli mentioned on the y axis. Darkened boxes (brown) denote the presence of a particular compound represented on the x axis. Compounds with a maximum molecular weight of 200 are considered in the data. For the complete list of compounds, please see extended Table S1. *n* = 3–5 chromatograms per stimulus category. (B) A representative chromatogram trace of lignin headspace collected with SPME showing a peak corresponding to guaiacol. (C) Preference indices representing behavioral valence of lignin when tested with four *Drosophila* species. Highlighted violin plots denote significantly different preferences when compared to neutrality. ∗*p* < 0.05. Unpaired parametric test followed by Welch’s correction. (D) Oviposition preference of *D. virilis* when presented with lignin. Highlighted violin plot denotes significantly different preference when compared to neutrality. ∗*p* < 0.05. Unpaired parametric test followed by Welch’s correction. (E) Preference indices representing the choice of multiple *Drosophila* species when tested between guaiacol (10^−4^ v/v) and control. Highlighted violin plots denote significantly different preferences when compared to neutrality. ∗*p* < 0.05. Unpaired parametric test followed by Welch’s correction. (F) Oviposition preference of *D. virilis* when presented with guaiacol identified as an attractant in previous experiments. The highlighted violin plot indicates a significant difference between the indices and the neutral line (zero). Unpaired parametric test followed by Welch’s correction.

Although the association between flies from the *virilis* group and slime fluxes has been known for half a century, there is a severe dearth of knowledge when it comes to our understanding of ecologically relevant ligands for *D. virilis.* To resolve this, next, we analyzed the collected slime flux by performing liquid injections for samples collected from the field site as headspace volatile collection (solid-phase microextraction [SPME]) was not possible in the field. Liquid injections of the filtered hexane/methanol fractions revealed more than 120 chemically diverse compounds of different biological origins (Table S1; Figure 2A). We found several long-chain, high-molecular-weight compounds (>200 MW), as well as some pheromones such as 9-tricosene that might be attributed to the presence of fly larvae within the collected flux. Under natural conditions, slime fluxes are known to occur cryptically inside wounds in tree barks. Such cracks are primarily a result of abiotic stress caused during winter and subsequent thawing.^22^ Tree cracks are actively repaired by rapid synthesis and deposition of lignin, a building block of the tree bark.^23^ Additionally, *D. virilis* adults have also been collected from breweries, where they are mostly associated with barrels, and also from stored wooden logs.^24^ All of these previously reported collection substrates involve wood undergoing complex conversions which, in turn, are known to release lignin-associated phenolics^25^ and therefore, compelled us to check if lignin alone has any odor and has any behavioral valence in *D. virilis* when tested alone. Our experiments with pure lignin powder revealed lignin as a strong behavioral cue that triggered both attraction and oviposition in *D. virilis* (Figures 2C and 2D). Interestingly, *D. immigrans* was also found to be attracted to pure lignin powder while *D. pseudoobscura* and *D. melanogaster* did not show any significant behavior toward lignin (Figure 2C). These findings initially perplexed us as lignin is a macromolecule with low vapor pressure. Hence, we checked the headspace of synthetic lignin powder only to find one major peak of 2-dimethoxyphenol (henceforth called guaiacol) (Figure 2B). Coincidently, guaiacol was also present in the natural slime flux site (Figure 2A, dotted red box) and guaiacol along with other phenolics is a known breakdown product of lignin.^26^

Next, we tested the valence of guaiacol across 12 *Drosophila* species in an ecologically relevant concentration (10^−4^ v/v) in a binary choice assay. The tested species panel consisted of four species from the *virilis* group while the remaining species represented flies known to inhabit different ecological niches such as rotting fruits, vegetables, etc^8^^,^^27^ (Figure 2E). Interestingly, only *D. virilis* and *D. ezoana*, both belonging to the *virilis* group, were strongly attracted to guaiacol at ecologically relevant concentrations (10^−4^ v/v). We also observed a strong dose-dependent attraction as high concentration of guaiacol (10^−2^ v/v) was observed to be significantly aversive in *D. virilis* in line with a previous report^28^ (Figure S1A). Moreover, guaiacol appears to act only as an attractant in *D. virilis*, as it did not contribute to a positive oviposition behavior when tested at the same concentrations (Figure 2F). It is intriguing to find that *D. immigrans* is attracted to lignin but not to guaiacol (Figures 2C and 2E) suggesting the use of other lignin-derived compounds as attractants in this species. In conclusion, we report a chemical database relevant to the natural life history of *D. virilis* and identify guaiacol as a strong attractant for *D. virilis.* We propose that, in the wild, the breakdown phenolic components of lignin such as guaiacol serve as host cues signifying the breakdown of tree bark and thereby the potential presence of slime flux.

### *Dvir*Or49b is narrowly tuned to detect guaiacol and other phenolics

**Figure 3:**
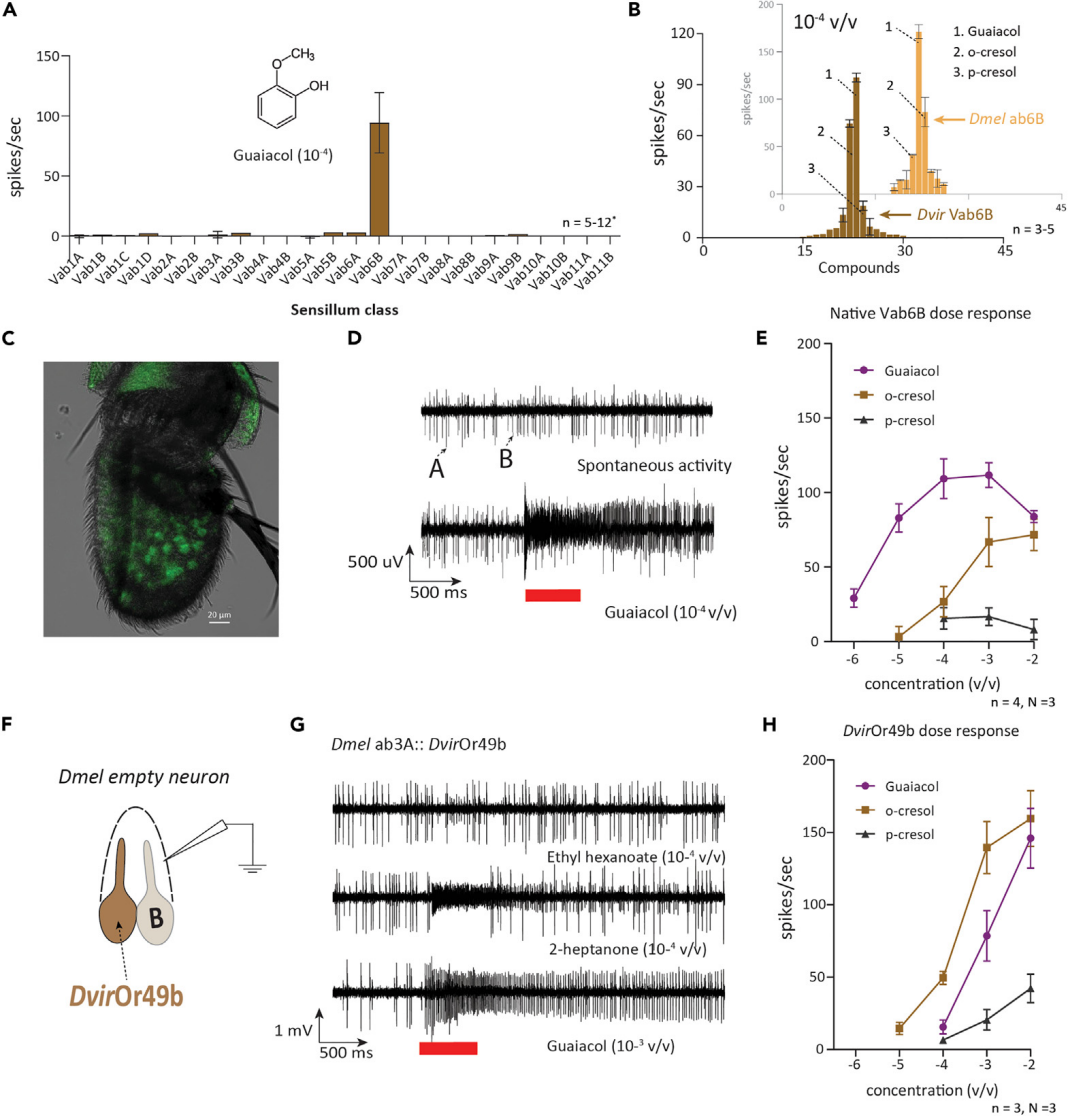
*Dvir*Or49b is narrowly tuned to phenolics primarily to guaiacol and *o*-cresol (A) Single sensillum recordings (SSR) from 11 antennal basiconic types identified previously.^28^ The sensillum classes were challenged with known diagnostic odors^28^ and guaiacol diluted in hexane (10^−4^ v/v). Error bars represent SEM. (B) Tuning curve of Vab6B, where a panel of 45 odors (43 odors in 10^−4^ v/v dilution plus two controls as hexane and air) was used. Tuning curve panel (orange) in the background represents recordings from the ab6B OSN class in *D. melanogaster* to the same odor panel. While the panel in the front (brown) represents responses from Vab6B OSNs in *D. virilis*. Error bars represent SEM. Tuning curve comparison between species revealed conserved response spectra and guaiacol and *o*-cresol as the best ligands. Red bar at the bottom represents stimulus delivery period of 500 ms. (C) *In situ* hybridization experiments to demonstrate expression of *Dvir*Or49b in the antenna of *D. virilis*. (D) Representative trace of SSRs from the Vab6 sensillum type. Upper: the spontaneous activity showing the larger A neuron compared to the smaller B neuron. Lower: a strong response to guaiacol (10^−4^ v/v dilution). (E) Dose response SSR experiment in Vab6B neurons to methylphenols. Error bars represent SEM. (F) Schematic representation of *D. melanogaster* empty neuron system used to express and deorphanize *Dvir*Or49b. (G) Representative traces from the homozygous *D. melanogaster* fly having a genotype (+; Df(2L)Or22ab,Gal4-Or22ab/Df(2L)Or22ab,Gal4Or22ab; UAS-*Dvir*Or49b/UAS-*Dvir*Or49b). Upper two panels show responses to ethyl hexanoate and 2-heptanone, respectively, demonstrating successful elimination of the native *Dmel*Or22a/b. Lower: a strong response to guaiacol. Red bar at the bottom represents stimulus delivery period of 500 ms. (H) Dose-response experiment to test the sensitivity of the *Dvir*Or49b expressed in *D. melanogaster* empty neuron system to methylphenols. Error bars represent SEM.

Next, we focused on identifying olfactory sensory neurons (OSNs) and, eventually, odorant receptor/s (OR/s) involved in the detection of the phenolics. We recently established the specificity of OSNs present in the antenna of three closely related species from the *virilis* group and were able to identify 11 sensillum classes based on odor response profiles.^28^ As guaiacol was the dominant compound in our previous chemical analyses and had a strong behavioral effect, we first sought to identify the guaiacol detection pathway in *D. virilis*. We screened the previously reported 11 antennal basiconic sensillum types with a panel of 43 chemically diverse compounds, including guaiacol, and identified an OSN type (Vab6B) narrowly tuned and strongly responding to guaiacol^28^ (Figures 3A, 3B, and 3D). We also challenged the known ab6 sensillum type in *D. melanogaster* using the same odor panel and observed a near identical tuning pattern between the two species^29^^,^^30^ (Figure 3B). This indicated a possible involvement of a *D. virilis* OR homologous to the OR known to be expressed in the ab6B OSN type in *D. melanogaster* (Or49b), which is narrowly tuned to phenolics (specifically methylphenols).^31^ Consequently, we sought to deorphanize the orthologous *Dvir*Or49b and started by confirming the expression of *Dvir*Or49b in the antenna of *D. virilis* using *in situ* hybridization. A clear expression pattern of *Dvir*Or49b could be observed (Figure 3C). Next, we proceeded to perform single sensillum recordings from the native Vab6B type OSN, where dose-response experiments revealed maximum sensitivity to guaiacol followed by *o*-cresol (also known as 2-methylphenol) (Figures 3D and 3E). We took advantage of the “latest” empty neuron system in *D. melanogaster* generated by a CRISPR-Cas9-based knockin strategy to deorphanize *Dvir*Or49b^32^ (Figure 3F). Subsequently, we challenged this heterologously expressed OR with the same panel of 43 odorants used in our initial screen and observed that *Dvir*Or49b responded to an identical set of ligands (guaiacol, *o*-cresol and *p*-cresol) as compared to responses in its native environment in the Vab6B OSN (Figures 3G and S1B). However, the sensitivity was somewhat lower and *o*-cresol was observed to be the best ligand followed by guaiacol. The same pattern was reflected in the dose-response experiment, where the heterologously expressed OR was highly sensitive to *o*-cresol followed by guaiacol, a pattern showing a ligand rank exchange compared to its native environment (Figure 3H).

The empty neuron system is a highly efficient method to deorphanize ORs. However, the responses obtained have, in some cases, been observed to be influenced by the lack of factors present in the native OR environment. For instance, accessory proteins such as sensory neuron membrane proteins (SNMPs; for pheromones) and odorant-binding proteins (OBPs) have been shown to affect response dynamics.^33^^,^^34^^,^^35^^,^^36^ However, taken together, our experiments reveal *Dvir*Or49b as the tuning receptor for guaiacol and *o*-cresol in *D. virilis*.

### *D. virilis* is attracted to *o*-cresol, a major phenolic component from beaver castoreum

**Figure 4:**
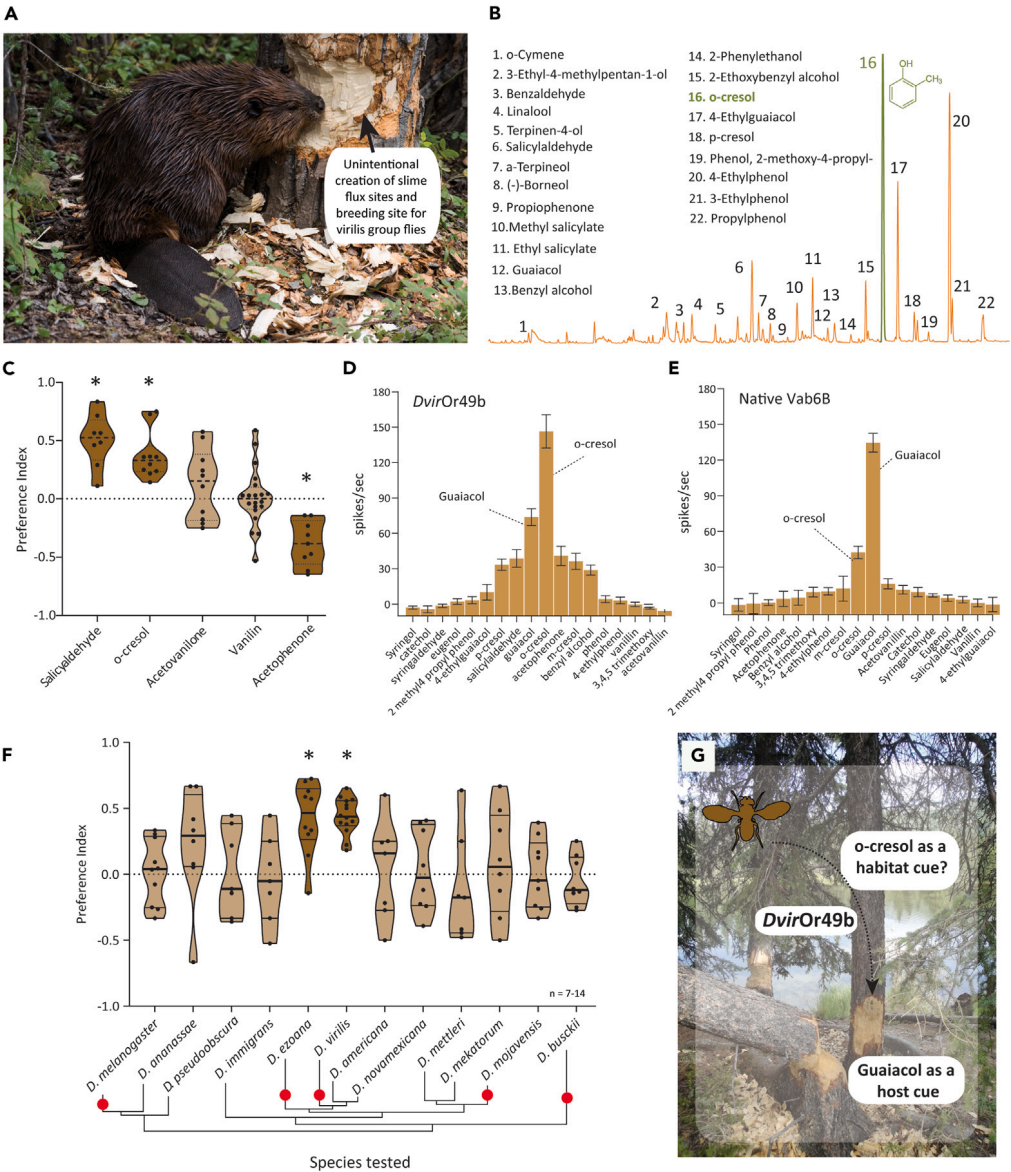
*o*-cresol, component found only in beaver castoreum, is a specific attractant for two species from the *virilis* group (A) A beaver chewing the bark of a tree. A representative pictorial depiction of a hypothesis suggesting indirect commensalism between beaver and species from the *virilis* group^18^ (Photo credits: National Park Service, US Department of the Interior). (B) A chromatogram showing the chemistry of beaver castoreum tincture and compounds identified from it. (C) Preference indices representing choice of *D. virilis* flies when tested between lignin/lignin fermentation-derived individual compounds and control. Highlighted violin plots denote significantly different preference when compared to neutrality. ∗*p* < 0.05. Unpaired parametric test followed by Welch’s correction. (D) Tuning curve of the *Dvir*Or49b expressed in the empty neuron system when presented with phenolics identified from beaver castoreum (10^−3^ v/v dilution). (E) Tuning curve of the native Vab6B OSN class when presented with phenolics identified from beaver castoreum (10^−3^ v/v dilution). (F) Preference indices representing choice of multiple *Drosophila* species when tested between *o*-cresol (10^−4^ v/v) and control. Highlighted violin plots denote significantly different preference when compared to neutrality. ∗*p* < 0.05. Unpaired parametric test followed by Welch’s correction. Filled red dots represent species where at least one sensillum class responding to *o*-cresol is known from the literature. (G) A schematic model based on results obtained in this figure and Figure 2 showing involvement of phenolics as attractants for *D. virilis* (Photo credits: National Park Service, US Department of the Interior).

Our deorphanization experiments revealed guaiacol and *o*-cresol as the two best ligands for *Dvir*Or49b. The source of guaiacol was already known to us. A search for the natural occurrence of *o*-cresol revealed that this compound is one of the major components of the beaver scent gland excretions (also called castoreum) along with secretions from mammals and arthropods.^37^^,^^38^^,^^39^^,^^40^ Interestingly, an old hypothesis predicts indirect commensalism between beavers (*Castor* spp.) and flies from the *virilis* group.^18^ The geographical distribution of beavers and flies from the *virilis* group highly coincides in the temperate region of the world where a potential overlap between their habitats has been hypothesized as they are known to inhabit resources close to freshwater.^15^^,^^18^^,^^41^ The hypothetical interaction revolves mainly around the fact that damage of trees by beaver activity would give rise to potential slime flux sites and thereby unintentionally create beneficial breeding grounds for *virilis* species (Figure 4A). Although experimental testing of this hypothesis has never been done, the hypothesis holds potential as some of the best collections of wild *virilis* group flies have been reported when done over the water surface or along its edge.^12^ This is the exact range where beavers are active and deposit castoreum to mark their territories.^42^ Also, in a healthy forest, chance of finding wounded trees with slime fluxes is sparse and could be attributed mainly to abiotic factors such as cracks formed during winters.^9^ However, the frequency of such events and the availability of sites with an active slime flux at a given time are low. Previous reports describing the occurrence rate of natural slime fluxes and our own efforts to locate a flux-producing site clearly indicate the rarity of this event in nature.^9^^,^^10^ Hence, it is possible that the involvement of another player facilitating the continuous provision of slime flux-bearing sites would favor indirect commensalism.

Beaver castoreum is a complex chemical mixture and contains a large number of phenolics.^17^ We obtained commercially available castoreum tincture stored in ethanol (38% in ethanol) and found that castoreum (10^−2^ v/v) itself was attractive to *D. virilis* (Figure S1D). A subsequent chemical analysis of the tincture headspace using SPME revealed that the volatile composition was heavily dominated by phenolics, where *o*-cresol was one of the most dominant peaks (peak 16, Figure 4B). We also found salicylaldehyde, a compound generally found as a conversion product of salicylic acid present in the bark of willow (*Salix* sp.), which is one of the highly favored hosts of beavers.^17^ Lastly, beaver castoreum has been historically used as a source for extracting vanilla flavor as the secretion resembles the smell of vanilla due to the presence of compounds like vanillin and acetovanillone. Next, we tested if these compounds have a behavioral role in *D. virilis*. Interestingly, both salicylaldehyde and *o*-cresol were highly attractive in *D. virilis* (Figure 4C). Next, we challenged the *Dvir*Or49b, transiently expressed in the *D. melanogaster* empty neuron system, with a panel of 18 chemically similar phenolics and observed that this OR was narrowly tuned to guaiacol and *o*-cresol with a weaker response to salicylaldehyde (Figure 4D), but when the Vab6B OSN was tested in its native environment, it responded only to the first two compounds (Figure 4E). In conclusion, we find that *Dvir*Or49b responds to phenolics but is narrowly tuned to two structurally very similar methylphenols (guaiacol and *o*-cresol). *o*-cresol has been used as a part of odor panels used in single sensillum recording screening studies since the early 2000s.^29^^,^^31^ Moreover, at least four species display conserved sensillum type(s) involved in the detection of *o*-cresol^7^^,^^8^^,^^28^^,^^31^ (Figure 4F, filled red circles). This made us wonder if the behavioral valence of *o*-cresol is also conserved across the phylogeny and if our observations with respect to *D. virilis* were only a chance event. We tested the attraction behavior of multiple drosophilids spanning the phylogeny against the same concentration of *o*-cresol as tested for *D. virilis* (Figure 4F, 10^−4^ v/v). We found that only two out of the 12 species tested, *D. virilis* and *D. ezoana*, were highly attracted to *o*-cresol, while others were not (Figure 4F), mirroring the exact pattern observed in the case of guaiacol (Figure 2E). *D. americana* and *D. novamexicana* both belong to the *virilis* group yet displayed a neutral behavior toward *o*-cresol. First, *D. novamexicana* is known to inhabit warm regions (such as New Mexico, USA) that do not coincide with the beaver territories.^43^ Secondly, no sensillum type responding to *o*-cresol was found during the antennal screening in *D. novamexicana*,^28^ which supports our observations here. Lastly, similar to guaiacol, a dose-dependent shift in behavioral valence was observed in the case of *o*-cresol (Figure S1C). Taken together, our experiments investigate the sensory cues involved in the hypothetical beaver-*virilis* indirect commensalism and, from these directions, provide a possible olfactory background by demonstrating *o*-cresol (a compound found in beaver castoreum) as a strong attractant for *D. virilis*. Finally, based on our experimental results and identification of guaiacol and *o*-cresol as attractants in *D. virilis*, it can be hypothesized in a broader context that *o*-cresol could be used as a habitat cue,^44^ while guaiacol would be a direct indication of a suitable host substrate cue by *D. virilis* (Figure 4G).

### Conclusion

We established the volatile headspace database (olfactome) of a slime flux site, a recognized ecological niche for species within the *virilis* group. Our findings underscore the pivotal role of lignin and various phenolic compounds, including guaiacol and *o*-cresol, influencing the behavior of *D. virilis*. Moreover, our experiments lend partial experimental support to a long-standing hypothesis proposing indirect commensalism between beavers and species from the *virilis* group. Taken together, our study furnishes a valuable olfactome and provides a robust foundation for future investigations involving species from the *virilis* group and insects inhabiting the broader forest ecosystem.

### Limitations of the study

It must be noted that the slime flux collected in this study was collected from a single birch tree while species from the *virilis* group are known to be associated with several other tree species including willow, aspen, etc. Therefore, it will be important in the future to collect slime fluxes from several tree species and to investigate if and how the chemical profile of slime flux varies across tree species. The ecology of several species from the *virilis* group is poorly understood. Hence, it would be greatly important to carry out fieldwork in the future and to shed light on the natural behaviors of these species. Moreover, it is possible that several other compounds found in the headspace of slime flux (apart from guaiacol) could serve as host cues in *D. virilis* and could be equally important in the life history of the species for host recognition. Here, we have proposed that guaiacol (detected by *Dvir*Or49b) serves as a host recognition cue in *D. virilis*. However, it is possible that the *Dvir*Or49b-mediated pathway is not the sole pathway involved in host recognition in *D. virilis*. Testing *Dvir*Or49b mutants with field-collected slime fluxes as well as identification of other attractants from the flux would be required to address this possibility. Lastly, it is intriguing to find that *o*-cresol (found in the beaver castoreum and detected by *Dvir*Or49b) acts as an attractant in the case of *D. virilis* and *D. ezoana* in light of the existing beaver-*virilis* indirect commensalism hypothesis. However, we cannot rule out the possibility that the *Dvir*Or49b-based pathway is primarily employed for detecting guaiacol while *o*-cresol detection could be due to structural similarity. It must be noted that castoreum is a complex stimulus composed of several compounds (such as methyl salicylate, 2-phenyl ethanol, 4-ethyl guaiacol, etc.), which have previously been reported as strong attractants in other *Drosophila* species. Finally, the castoreum stimulus used in this study is a tincture diluted in ethanol. Therefore, the observed attraction to the castoreum could be a result of a complex activation of several compounds and a result of multiple circuit interactions.

## Resource availability

### Lead contact

Further information and requests for resources and reagents should be directed to and will be fulfilled by the lead contact, Prof. Dr. Bill S. Hansson (hansson@ice.mpg.de).

### Materials availability

This study did not generate new unique reagents.

### Data and code availability

• All data reported in this paper will be shared by the lead contact upon request.
• This paper does not report original code.
• Any additional information required to reanalyze the data reported in this paper is available from the lead contact upon request.

## Acknowledgments

This study was supported by the 10.13039/501100004189Max Planck Society (R.S.-R., M.K., and B.S.H.), by Max Planck Centre next Generation Chemical Ecology (V.P.M., M.K., and B.S.H.), and the International Max Planck Research School (IMPRS) at the Max Planck Institute of Chemical Ecology (V.P.M.). We thank Silke Trautheim, Roland Speiss, Ibrahim Alali, and Manal Alali for their help with maintaining fly stocks. We thank Angela Lehman and Kerstin Weniger for their technical assistance in the chemistry lab. We thank Swetlana Laubrich for the administrative assistance. We thank Emily Puckett for her help with fungal strain experiments. Images in the graphical abstract were obtained from BioRender.com.

## Author contributions

V.P.M., M.K., and B.S.H. conceived the project. V.P.M., M.K., and B.S.H. designed the experiments. V.P.M. conducted most of the experiments, created the figures, analyzed the data, and wrote the first draft of the manuscript. R.S.-R. generated the transgenic fly and conducted genetic crossings and *in situ* hybridization experiments. V.P.M. was supervised by M.K. and B.S.H. V.P.M., R.S.-R., M.K., and B.S.H. discussed the results. All authors wrote the final version of the manuscript.

## Declaration of interests

The authors declare no competing interests.

## Declaration of Generative AI and AI-assisted technologies in the writing process

During the preparation of this work, the author(s) used ChatGPT in order to check grammar and to ensure that the word limit rules were followed. After using this tool, the author(s) reviewed and edited the content as needed and take full responsibility for the content of the publication.

## STAR★Methods

### Key resources table

**Table:** 

REAGENT or RESOURCE	SOURCE	IDENTIFIER
**Antibodies**		

anti-digoxigenin alkaline phosphatase-conjugated antibody	Merck	11093274910

**Chemicals, peptides, and recombinant proteins**		

For information regarding chemicals used in this study, please refer to Table S3	Multiple	

**Critical commercial assays**		

For information regarding commercial assays used in this study, please refer to STAR Methods		

**Experimental models: Organisms/strains**		

*Drosophila ananassae*		14024–0371.11
*Drosophila melanogaster Canton S*	Hansson lab strain	
*Drosophila busckii*		13000–0081.00
*Drosophila mojavensis*		15081–1352.10
*Drosophila virilis*		15010–1051.00
*Drosophila mercatorum*		15082–1521.00
*Drosophila immigrans*		15111–1731.00
*Drosophila pseudoobscura*		14011–0121.00
*Drosophila americana*		15010–0951.00
*Drosophila mettleri*		15081–1502.11
*Drosophila ezoana*		E−15701
*Drosophila novamexicana*		15010–1031.08

**Oligonucleotides**		

*Dvir*Or49b-Forward		5′ ATGCTTGAGGATATACA ATTCATTTACATGAACGTAC 3′
*Dvir*OR49b-Reverse		5′ TTAACCGTAAATACGTT TAAGTACCGTGAAAAATGTG 3′

**Software and algorithms**		

NIST library 2.3.	Software	https://chemdata.nist.gov
GraphPad-Prism 9.1.1	Software	https://www.graphpad.com/scientific-software/prism/
AutoSpike32 software 3.7 version	Software	http://www.ockenfels-syntech.com
Adobe Illustrator and Photoshop	Software	https://www.adobe.com
BioRender	Software	https://www.biorender.com
XCMS	Software	https://xcmsonline.scripps.edu
ChatGPT	Software	https://chatgpt.com

**Other**		

Beaver castoreum tincture (38% in ethanol)	Etsy.com	N/A

### Experimental model and study participant details

Wild-type *Drosophila* flies were used in this study and a detailed description of their original sources and stock numbers are listed in Supplementary Table 1 and listed in the key resources table. Flies were reared on different food media listed in Supplementary Table 2 and were maintained at 12:12 h light: dark cycle at 23°C and 40% relative humidity. A description of newly generated transgenic flies has been given separately. Groups of both male and female flies were used for testing attraction behavior while only females were used for testing oviposition behavior and for electrophysiology experiments. This study on fruit flies was performed in Germany, where research on invertebrates does not require a permit from a committee that approves animal research.

### Method details

#### Chemical stimuli

All chemicals used in this study were purchased with the highest purity possible. A list of all odorants used along with their suppliers is available in Supplementary Table 3. Odorants were diluted in either hexane or acetone for the single sensillum recording experiments to screen the *D.virilis* antenna and appropriate solvent controls were subtracted from final responses. Mineral oil was used as a solvent for testing compounds used in behavioral bioassays. Lignin was used as a pure powder (CAS: 8068-05-1). Castoreum was bought as a tincture stored in 38% ethanol (source: Etsy) and diluted in distilled water for behavioral bioassays.

#### Slime flux chemical analysis (liquid injection-GC-MS)

Samples were obtained from the field and were added with 1 mL of hexane or methanol and kept overnight at 4°C with constant shaking. Subsequently, the samples were filtered and stored at −20°C until further analysis. Solvent extracts (1 μL) were injected in a gas chromatograph equipped with a non-polar HP5 (Agilent 5975) or a polar (wax) column (Agilent 19091S-433U, 30 m length, 250 μm diameter and 0.25 μm film thickness, Agilent technologies) with helium as carrier gas and connected to a mass spectrometer. The temperature of the oven was held at 40°C for 3 min, increased by 5 °C min^−1^ to 260°C and 280°C in the wax and HP5 columns respectively. The final temperature was held for 5 min. The MS transfer line was held that 260°C, the MS source at 230°C and the MS quad at 150°C. Mass spectra were scanned in EMV mode in the range of 29 mz^−1^ to 350 mz^−1^. For liquid injection samples, the same program was used except the temperature increase ramp was slower and held at 5°C per minute. Chromatograms were visualized using Enhanced data analysis software (Agilent Chemstation, Agilent technologies) and manually analyzed using NIST library 2.3. (https://chemdata.nist.gov). A principal component analysis of all chromatograms was generated using an online software called XCMS version 3.7.1.^45^

#### Bioassays

##### Oviposition and preference assay

Wild-type flies were used for oviposition and preference bioassays described previously elsewhere.^8^^,^^28^ As *D. virilis* are known to reach sexual maturity after an age of ∼6 days,^46^ all experiments used flies more than 10 days old. 10 female flies along with 3 males were used for oviposition experiments conducted in salad boxes (transparent plastic boxes, 500 mL volume, with 10 ventilation holes punctured with forceps, https://www.pro-pac.de/gb/products/salad-boxes/500507/). Flies were sorted one day prior to the experiment (8^th^ day) using CO_2_ pads and supplied with yeast granules *ad libitum* overnight. A central hole was punctured in 0.25% agarose plates so as to make a cavity of ∼8∗9 mm (diameter ∗ height). Stimuli were put in this cavity and covered with a filter paper (Rotilabo-round filters, type 601A, Carl Roth GmbH, Germany) of ∼10 mm diameter. To ensure the presentation of only olfactory stimuli, a filter paper covered the stimulus (10 μL of a test odorant dissolved in mineral oil was used in the case of experiments using individual odorants) in all experiments. Two plates (test and control) were ∼1 cm apart in salad boxes. Experiments generally began around 1100 h and were terminated around the same time. Eggs were manually counted after 48 h with a 16L: 8D photoperiod during testing. Oviposition and preference indices were calculated as (T-C)/(T + C) where T represents number of eggs (or flies caught) on the test plate/trap while C represents the same on control plate/trap. For preference index, traps were manufactured by attaching pink paper cones on plastic vials and 300 μL of test or control compound were pipetted into two cut Eppendorf tubes (E−1015) caps. A group of 30 flies was used for preference assays as described previously.^28^ Attraction to castoreum was tested in large cages (∼50 cm^3^, BugDorm-44545 F, https://shop.bugdorm.com/distributors.php) with a group of 50 flies per replicate.

##### Electrophysiology

Single sensillum recordings (SSR) were performed by following a protocol previously described in detail elsewhere.^47^ Generally, 8–10 days old female flies were used for the experiment. In short, flies were held immobile in a pipette tip in a way that the head capsule was accessible to the experimenter. The third antennal segment was extended and held immobile on a coverslip using a glass capillary so that the sensilla on the medial side were accessible. An electrochemically sharpened tungsten electrode was inserted in the base of a sensillum (recording electrode) while another electrode was inserted in the eye (reference electrode). The potential difference between these two electrodes was amplified and changes in the spontaneous activity post stimulation were recorded. Signals were amplified (Syntech Universal AC/DC Probe), sampled (96,000/s) and filtered (3 kHz High-300 Hz low, 50/60 Hz suppression) using a USB-IDAC. Guaiacol was diluted in hexane and tested at 10^−4^ conc. (v/v) for an experiment involving antennal screening to identify sensillum class(es) responding to the same. Diluted odorants were pipetted in an odor cartridge as described previously^47^ and the same cartridge was used not more than 3 and 5 times for dose response and antenna screening experiments respectively unless stated otherwise. For dose-response experiments, concentrations were tested in an ascending order (lowest to highest) with an interval of at least 1 min in between two deliveries. For, experiments involving multiple phenolics, the compounds were diluted either in hexane or in acetone to a concentration of 10^−3^ v/v. Compounds dissolved in hexane are salicylaldehyde, 2-methy-4-propyl phenol, eugenol, acetophenone, benzyl alcohol, guaiacol, p/m/o-cresol. While acetone was used as a solvent for syringol, syringaldehyde, phenol, acetovanillone, vanillin, catechol, 4-ethylphenol, 4-ethylguaiacol, 3-4-5-methoxy phenol.

##### Cloning of *D. virilis* Or49b receptor and transgenic expression in *D.* *melanogaster* ab3A neuron

To clone the full-length coding sequence of *Drosophila virilis* Or49b (*Dvir*Or49b), total antennal RNA from approximately 100 *D.* *virilis* flies was prepared and cDNA was synthesized using the Superscript III Reverse Transcriptase kit (Thermo Fisher Scientific, Schwerte, Germany). The gene of *Dvir*Or49b was amplified using PCR with the following gene specific primers: *Dvir*Or49b-Forward: 5′ ATGCTTGAGGATATACAATTCATTTACATGAACGTAC 3’; *Dvir*OR49b-Reverse: 5′ TTAACCGTAAATACGTTTAAGTACCGTGAAAAATGTG 3’. The resulting construct of the PCR reaction (1116bp) was purified and T:A cloned into pCR8/GW/TOPO (Thermo Fisher Scientific, Schwerte, Germany). Sanger sequencing confirmed the sequence (Eurofins Genomics Germany GmbH and the Department of Insects Symbiosis, Max Planck Institute for Chemical Ecology, Jena). The transfer of the construct to the destination vector pUASg.attB (kindly provided by J.Bischof, FLYORF Injection, Zürich, Switzerland) was completed as previously described.^48^ Subsequently, germline transformation of *D. melanogaster* with the prepared plasmid was performed by FlyORF Injection (Zurich, Switzerland) using the PhiC31 integration system. The vector was inserted into chromosome III, fly strain ZH-86Fb to produce the genotype +; +; UAS-*Dvir*Or49b/TM6b. Transgenic *D. melanogaster* flies were bred on conventional cornmeal agar medium under a 12h light and 12h dark cycle at 25°C and 70% humidity. To generate the experimental fly line: +; Df(2L)Or22ab,Gal4-Or22ab/Df(2L)Or22ab,Gal4Or22ab; UAS-*Dvir*Or49b/UAS-*Dvir*Or49b which expresses *Dvir*Or49b under regulation of Or22a in an Or22a and Or22b deleted background, following fly lines were used: (+; +; UAS-*Dvir*Or49b/TM6b), (yw; BL/Cyo; TM2/TM6b) and (+; Df(2L)Or22ab,Gal4Or22ab/Cyo; TM2/TM6b). The parental line (yw; BL/Cyo; TM2/TM6b) was obtained from Bloomington Stock Center no. BL3704) and parental line (+; Df(2L)Or22ab,Gal4Or22ab/Cyo; TM2/TM6b) was kindly provided by Prof. J.R. Carlson (Dept. of Molecular Cellular and Developmental Biology, Yale University).

##### Generation of antisense Riboprobes for fluorescence *in situ* hybridization (FISH)

For the antisense probe, the gene *Dvir*Or49b was amplified by PCR and cloned into pCRII-TOPO TA-Vector (Thermo Fisher Scientific, Schwerte, Germany) containing T7/Sp6 binding sites. To generate the Biotin-labelled antisense probes from the plasmid the T7/Sp6 RNA transcription system (Roche Diagnostics, Mannheim, Germany) was used as recommended by the manufacturer. For the tissue preparation flies were anesthetized under CO_2_ and the heads were cut off. Subsequently, the back of the heads was opened for better penetration of the fixative solution. The heads were immediately transferred into a fixing solution (4% paraformaldehyde in 0.1 M NaHCO_3_, pH 9.5) for 2h while rotating at 6°C. Next, fixed heads were washed twice for 5 min in PBS (0.85% NaCl, 1.4 mM KH_2_PO_4_, 8 mM Na_2_HPO_4_, 0.03% Triton-100, pH7.4), incubated for 10 min in HCl (0.2M HCl, 0.03% Triton-100) and washed again in PBS for 1min. Then, heads were pre-hybridized for 1h at 55°C in a hybridization solution [50% formamide, 5x SSC (0.75M NaCl, 0.075M Na_3_C_6_H_5_O_7_, 0.03% Triton-100, pH 7.0), 1x Denhardts reagent, 50μg/ml RNA yeast, 1% Tween 20, 0.1% Chaps, 5mM EDTA pH8.0, 0.2 mg/mL herring sperm]. Subsequently, heads were covered and incubated with hybridization solution containing the Biotin tagged antisense RNA probe for 36–72 h rotating at 55°C. The heads were then washed 4x for 15 min at 60°C with 0.1 SSC. This was followed by a blocking step carried out for 2 hrs at room temperature in 1% Blocking solution (Roche Diagnostics, Mannheim, Germany) in Tris-buffered Saline (TBS; 100 mM Tris, 150mM NaCl, 0.03% Triton-100, pH 7.5). Later, the antibody incubation was followed by addition of anti-digoxigenin alkaline phosphatase-conjugated antibody in 1:500 dilution in the blocking solution. The mix was incubated for 48 h while shaking at 6°C. Finally, heads were washed 5 times for 10 min with TBS 0.05% Tween 20. Visualization of the biotin-labelled probes was done with the TSA fluorescein system kit (Akoya Bioscience, Marlborough, USA). Incubation of heads with biotin-binding streptavidin conjugated to horseradish peroxidase and incubation with fluorescein-conjugated tyramides were conducted for 17 h at 6°C. The step was followed by a washing step where heads were rinsed for at least 3 times for 5min in TBS, 0.05% Tween 20. Finally, the heads were rinsed with water and the antennae were dissected and mounted on a coverslip with spacer in Vectashield (Vector Laboratories, Newark, USA). Mounted antennae were analyzed with a confocal LSM 780 laser-scanning microscope (Carl Zeiss Microscopy, Jena, Germany). Confocal image z stack was acquired from antennae in green fluorescence along with the transmitted light channels.

### Quantification and statistical analysis

Statistical analyses were performed using GraphPad-Prism 9.1.1 (https://www.graphpad.com/scientific-software/prism/). SSR traces were analyzed using AutoSpike32 software 3.7 version (Syntech, NL 1998). Changes in action potential (spike count) were calculated by subtracting the number of spikes 1 s before (spontaneous activity) from those elicited during 1 s after the onset of the stimulus. For behavioral data analyses, data was first tested for normal (Gaussian) distribution using Shapiro-Wilk normality test (significant = 0.05). Most of the data was observed to be normally distributed. For testing behavioral significance between two groups or between test and zero, unpaired parametric t-test with Welch’s correction was performed. For multiple comparisons between normally distributed groups, ordinary one-way ANOVA with multiple comparisons was performed. In the case of nonnormal distribution, non-parametric ANOVA with Kruskal-Wallis post hoc test was performed. Statistical details can be found in the legends of respective figures. Graphs were generated using GraphPad Prism 9.1.1. and figures were constructed and processed with Adobe Illustrator CS5 and Adobe Photoshop (Adobe system Inc.).

## Supplemental information

Document S1. Figure S1, Supplementary Tables S1–S3, and Data S1

Table S1. List of chemical compounds identified from natural slime flux site and from the headspace of synthetic lignin powder, related to the main figure 2
