# Supplementary material for: Phenolics as ecologically relevant cues for slime flux breeding Drosophila virilis
Source: iScience. 2024 Oct 18;27(11):111180. doi: 10.1016/j.isci.2024.111180 (PMC11567934; doi:10.1016/j.isci.2024.111180)
Supplement: Document S1. Figure S1, Supplementary Tables S1–S3, and Data S1 [file mmc1.pdf]

iScience, Volume *27*

Supplemental information

Phenolics as ecologically relevant cues for slime flux breeding *Drosophila virilis*

Venkatesh Pal Mahadevan, Regina Stieber-Rödiger, Markus Knaden, and Bill S. Hansson

Supplementary tables and figure

Supplementary Figure 1: Behavior and electrophysiology data related to main Figures 2-4

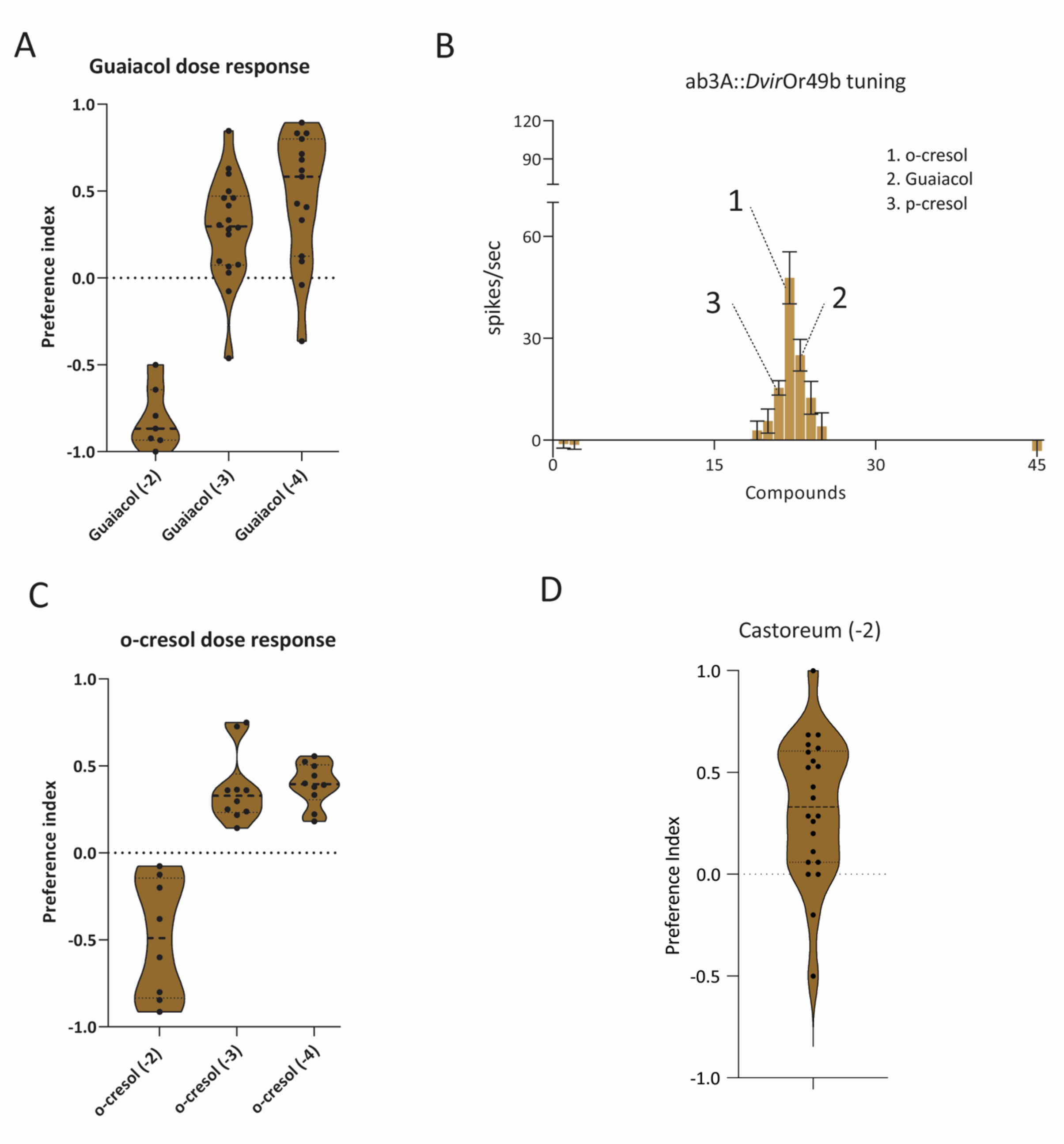

Supplementary figure 1 legends

A. A dose dependent attraction to guaiacol in *D. virilis.* Preference indices representing choice of *D. virilis* flies when tested between guaiacol and control. Highlighted violin

plots denote significantly different preference when compared to neutrality. * $p < 0.05$. Unpaired parametric test followed by Welch's correction (related to figure 2C)

B. Tuning curve of the transiently expressed *Dvir*Or49b in the *D. melanogaster* empty neuron system with an odor panel (10^{-4} v/v). Error bars indicated SEM. related to figure 3G)

C. A dose dependent attraction to o-cresol in *D. virilis.* Preference indices representing choice of *D. virilis* flies when tested between o-cresol and control. Highlighted violin plots denote significantly different preference when compared to neutrality. * $p < 0.05$. Unpaired parametric test followed by Welch's correction. (related to figure 4F)

D. Attraction to castoreum in *D. virilis.* Preference indices representing choice of *D. virilis* flies when tested between castoreum (10^{-2} v/v) and water. Highlighted violin plots denote significantly different preference when compared to neutrality. * $p < 0.05$. Unpaired parametric test followed by Welch's correction. (related to figure 4B)

Supplementary table 1: A list of all *Drosophila* species used in the study related to the STAR methods. The species were maintained in the laboratory for several generations. However, these species came originally from either Kyoto stock center (KC) or from the National Drosophila Species Stock Center at Cornell University (CU)

Species	food	stock number/ source
Drosophila ananassae	normal food	14024-0371.11
Drosophila melanogaster Canton S	normal food	
Drosophila busckii	normal:banana:wheat food (2:1:0.5 ratio)	13000-0081.00
Drosophila mojavensis	normal	15081-1352.10
Drosophila virilis	normal food	15010-1051.00
Drosophila mercatorum	normal food	15082-1521.00
Drosophila immigrans	normal food	15111-1731.00
Drosophila pseudoobscura	normal food	14011-0121.00
Drosophila americana	normal food	15010-0951.00
Drosophila mettleri	normal food	15081-1502.11
Drosophila ezoana	normal food	E-15701
Drosophila novamexicana	normal food	15010-1031.08

Supplementary table 2: A detailed composition of fly food types used in this study related to the STAR methods.

**Normal food**	unit	500 ml
treacle	g	59
brewer's yeast	g	5.4
Agar	g	2.1
Polenta	g	47
propionic acid	ml	1.2
Nipagin 30%	ml	1.65

**Banana food**	unit	
Agar	g	85
Yeast	g	165
Methyl paraben	g	13.4
Blended bananas	g	825
Karo syrup	g	570
Liquid malt extract	g	180
100% ethanol	ml	134
water	L	6

**Wheat food**	unit	
Semolina (corn based)	g	50
Wheatgerm	g	50
Sugar	g	50
Dry yeast	g	40
Agarose	g	8
propionic acid	ml	5
Methyl paraben	ml	3.3
water	L	1

Supplementary table 3: **List of odorants used for single sensillum recording experiments related to the STAR methods.** Abbreviations used: Sigma-Aldrich (Steinheim, Germany): SA, Acros Organics B.V.B.A.: AO, Fluka: F, Alfa Aesar: A.A, ABCR GmbH: ABCR, TCI chemicals:TCI. ROTH: RO and institute stock: Stock

	Odorant	CAS no.	Supplier : Catalogue number
1	Hexane	110-54-3	TCI: S0279
2	Ethyl acetate	141-78-6	SA: 270989
3	Ethyl lactate	97-64-3	SA: W244015
4	CO_2	Mouth aspiration	
5	Methyl salicylate	119-36-8	SA: M6752
6	Methyl acetate	79-20-9	F: 45999
7	Ethyl-3-hydroxybutyrate	5405-41-4	AO: 118540250
8	ethyl hexanoate	123-66-0	SA: 148962
9	2-heptanone	110-43-0	SA: W254401
10	E2-hexanal	6728-26-3	SA: 132659
11	geosmin	16423-19-1	SA: UC18
12	geranyl acetate	105-87-3	Stock (originally SA: 173495)
13	pentyl acetate	628-63-7	SA: W504009
14	1-octen-3-ol	3391-86-4	SA: W280518
15	guaiacol	90-05-1	SA: W253200
16	ethyl benzoate	93-89-0	SA: E12907
17	Ethyl crotonate	623-70-1	SA: 16794-0
18	acetoin	513-86-0	SA: W200808
19	linalool	126-91-0	SA: 74856
20	2 phenyalcohol	60-12-8	SA: 77861
21	benzyl butyrate	103-37-7	Stock (originally A.A: B24241)
22	2-butanone	78-93-3	SA: W217018
23	ethyl butanoate	105-54-4	SA: E15701
24	isopropyl benzoate	939-48-0	Stock (originally ABCR: AB137185)
25	Dimethyl disulphide	624-92-0	SA: 528013
26	acetone	67-64-1	RO: 5025.5
27	methyl benzoate	93-58-3	SA: 18344
28	6-methyl-5-helpten-2-one	110-93-0	SA: W270733
29	Hexyl acetate	142-92-7	SA: 10815-4

30	Isopentyl propionate	105-68-0	SA: W208205
31	2-nonanol	628-99-9	SA: N30307
32	Isopentyl alcohol	123-51-3	SA: w205710
33	1-hexanol	111-27-3	F: 471402
34	2-methyl phenol	95-48-7	F: 60990
35	2-nonanone	821-55-6	SA: N30307
36	Isopentyl acetate	123-92-2	SA: 30696-7
37	4-methylphenol	106-44-5	SA: 61030
38	Acetophenone	98-86-2	Stock (originally SA: 42163)
39	methyl hexanoate	106-70-7	SA: W270806
40	propyl acetate	109-60-4	SA: 133108
41	citral	5392-40-5	SA: C83007
42	2,3-Butanediol	513-85-9	SA: B84904
43	nonanal	124-19-6	SA: W278220
44	phenol	108-95-2	Riedel-de Haën: 33517

Data S1: *Dvir*Or49b coding sequence related to Figure 3.

ATGCTTGAGGATATACAATTCATTTACATGAACGTACGCATCCTGCGCTTCTGGGCGCTGCTCTACGA
TAAAAATATGAAACGCTATTTCTGCATCACGCTGAGCATCGTCCATGTGCTCACCCAGCTGCTGTATA
TGTTCAGCACCAACGAGGGCATCACGGGCATTATACGCAACTCCTATATGCTGGTGCTTTGGATAAA
TACCATATTGCGGGCCTGTTTGCTGCTGTTCGATCAGGAGAGCTATATCCAGCTGATTGACAATGTG
AGAGCCTACTACCATGAGCTGGAACGTGTGAAGGATAATTATATTGAGCGACTCTTGGTCCAGCTCA
ATCGGCAGGGTCAGCTTATGGCACGCGGCAATCTATTCCTTGGCCTGCTCACCTGCATTGGCCTTTGT
CTATATCCGATTAGTTTCAATGAGAGAGTTATGCCCTTTGGCAGCAAAATACCGGGCGTGAATGAGT
ATGCCACGCCCACCTATCAAATTTGGTTCGTCTTGCAAGTGCTCATCATACCGATGGGCTGCTGCATG
TACATACCCTACACCAGTCTCTGTGTGGCCTTCATCATGTTTGGCATTGTGATGTGCAAATCGCTGCA
GCATCGTTTGCGTTGTCTGAGCCGCAGACCCCTCAGCAGGCAGCAGCTCTCGCAGGAAATAATCGA
ATGCATTATCTATCATCAGCGGATCATAGATTATGTGCAGACCATCAATAAGCTGACTACATATATAT
TTCTCGTGGAGTTTTTGGCCTTCGGTGCGCTGCTCTGCGCATTGCTCTTTATGCTCATATTGATGGAC
ACCACGGCACAGGTGGCCATTGTGAGTGCGTACATAAACATGATACTCGCCCAGATATTGGCCCTTT
ATTGGTATGCCAATGAGCTAAGGGAGCAGAACTTGGCCATTGCCGGGGCCGCGTTTGAAGCCGATT
GGTTTACCTTTGATCTATCACTGCGCAAAGATATACAATTTATGATGTTGCGTGCTCAGCGGCCAGCA
TCGATACTCCTCGGCAATATACGACCCATTACATTGGAGCTATTTCAGAATTTACTAAATACATCTTAC
ACATTTTTCACGGTACTTAAACGTATTTACGGTTAA
